# Beyond legalisation: why assisted dying needs global monitoring

**DOI:** 10.3389/ijph.2026.1609777

**Published:** 2026-08-11

**Authors:** Paola Sillitti

**Affiliations:** 1 Faculty of Biology and Medicine (FBM) and Faculty of Business and Economics (HEC), University of Lausanne, Lausanne, Switzerland; 2 Palliative and Supportive Care Unit, Lausanne University Hospital (CHUV), Lausanne, Switzerland

**Keywords:** assisted dying, data availability, international comparison, monitoring and evaluation, public health

**The IJPH series “Young Researcher Editorial” is a training project of the Swiss School of Public Health**.

Assisted dying, which refers to all legally sanctioned practices intended to hasten death, is available or actively debated in a growing number of jurisdictions across Europe, Africa, America, and Oceania. Seventeen countries allow some form of assisted dying at the national or subnational level [1, 2]; others are discussing legalization of such practices [3]. About 500 million people live in jurisdictions where assisted dying is permitted [2]. In these jurisdictions, the number of people accessing assisted dying has generally increased over time [1]. Demand may continue to grow as populations age, and the burden of chronic conditions increases.

Research and policy debates over assisted dying have long focused on whether assisted dying should be legalised, and under what ethical and legal conditions [4]. While these questions are still central, as assisted dying becomes established practice in more countries, the debate must expand to include the question of how assisted dying should be monitored and evaluated.

Monitoring should comprise more than counting deaths. It should also track who accesses assisted dying, through which decision-making pathways, under what clinical and social circumstances, with what procedural outcomes, and whether access or reporting differs across population groups. In practice, the development of assisted dying is diverse across jurisdictions. Terminology, eligibility criteria, decision-making procedures, and reporting requirements vary substantially across countries [1, 3, 5, 6], making the international landscape of assisted dying highly heterogeneous; systematic monitoring remains fragmented.

Very few jurisdictions (e.g., some states in the United States) publish detailed annual reports that describe assisted dying cases and include the demographic and socioeconomic characteristics of the person who accessed assisted dying. It is rare to track the whole procedure of assisted death, including time-to-death and medication used [7]. In Australia, reporting includes some information on the procedure of assisted death, including time-to-death, but no information on medication used or complications during the dying process [8, 9]. Colombia publishes some demographic and clinical information, but does not track the procedure of assisted death [10, 11]. As debates about assisted dying increasingly take place in settings where data infrastructure is less developed, we are faced with the question of how to develop internationally comparable monitoring and evaluation systems for assisted dying.

The case of Switzerland illustrates the need for and limitations of current monitoring systems. Switzerland has a long history of assisted dying and its distinctive civil model of assisted dying is often referenced internationally as a successful example of a non-medicalised assisted dying process [4]. Nevertheless, the country does not maintain a comprehensive national registry and publicly available data are often limited to basic demographic information. Detailed information on practices and access is largely unavailable, limiting both national evaluation and international comparison.

Even where data exist, differences in definitions and reporting standards make international comparisons difficult [1]. We need consistent and transparent data to evaluate the practice of assisted dying, determine whether safeguards function as intended, or whether access varies across populations. From a public health perspective, this lack of standardisation creates a major gap because public health relies fundamentally on measurement. To answer questions about safety, quality of care, and equity of access, we need reliable data. We need monitoring systems to maintain public trust and inform evidence-based policy debates. Without comparable data across jurisdictions, discussions about assisted dying risk relying on incomplete information, limiting our ability to empirically inform ethical deliberation and policy debates.

Nations and international organisations should prioritize developing more consistent monitoring systems. The first necessary step to making international data more comparable would be integrating assisted dying into international health classification systems such as the International Classification of Diseases (ICD). Once this specific categorisation exists, countries where assisted dying is legal can consistently and transparently record and report cases. This data can be used to improve governance, and facilitate quality measurement.

The second necessary step would be for jurisdictions to report a minimum set of standardised indicators, including demographic and socioeconomic characteristics, underlying diagnosis or condition, number and timing of requests and assessments, involvement of health professionals or other organisations, medication used and place of death. They could also collect data on time from administration to death and complications or unexpected events to evaluate the quality of the death experience for people who opt for an assisted death and their families. Including equity indicators would allow us to assess whether access differs systematically by socioeconomic position, diagnosis, geography, care setting, cultural background, or other relevant population characteristics.

More standardised reporting would facilitate international research. As the practice spreads across countries, comparable data will be essential to understanding the functioning of different regulatory models, the way practices evolve over time, how people experience assisted dying, and whether disparities in access emerge across socioeconomic or demographic groups. Researchers can then focus on how assisted dying is implemented, governed, and experienced in practice.

The debate over assisted dying must evolve. Legal and ethical discussions will remain central, but should be complemented by a stronger focus on monitoring, transparency, and evaluation. Establishing standardised data collection systems and integrating assisted dying into international health classifications is an important step toward better understanding these practices. As more nations integrate assisted dying into contemporary end-of-life care, it should become a public health priority to ensure careful monitoring and evaluation.

## Data Availability

The original contributions presented in the study are included in the article/supplementary material, further inquiries can be directed to the corresponding author.
